# Power struggles: Absolute vs. relative EEG power in developmental neuroscience

**DOI:** 10.1016/j.dcn.2026.101698

**Published:** 2026-02-26

**Authors:** Aislinn Sandre, Sonya V. Troller-Renfree

**Affiliations:** a Department of Psychology, University of Western Ontario, London, ON N6G 2V4, Canada; b Department of Human Development, Teachers College, Columbia University, New York, NY 10027, USA

**Keywords:** Developmental electroencephalography (EEG), Absolute power, Relative power, Methodology, Resting-state EEG

## Abstract

Resting electroencephalography (EEG) is a central tool for studying early brain function and development. Yet, a key methodological decision—whether to quantify spectral activity using absolute or relative power—remains inconsistently applied and theoretically underdeveloped. Absolute power indexes the raw amplitude of oscillatory activity, whereas relative power expresses each frequency band as a proportion of the total signal. Relative power is often assumed to control for non-neural variability (e.g., hair texture), but this assumption has rarely been evaluated, particularly during periods of rapid developmental change. This commentary integrates conceptual analysis with empirical examples from multiple pediatric samples to evaluate whether relative power is indeed less biased by common sources of non-neural variability. Across frequency bands, absolute and relative measures showed both convergence and divergence: higher-frequency activity (e.g., gamma) aligned across indices, whereas lower-frequency activity (e.g., theta) did not, suggesting distinct neurophysiological and developmental properties. Relative power dampened some amplitude-related effects (e.g., fatigue), but remained influenced by hair texture, affect, and time of day. Together, these findings indicate that relative power does not universally correct for non-neural or state-related variability but instead provides a complementary representation of spectral composition. We recommend that developmental EEG studies report and interpret both absolute and relative power, justify analytic choices, and account for biological and contextual covariates. Greater clarity and consistency in how these metrics are used will improve the interpretability, reproducibility, and developmental relevance of EEG findings.

## Highlights

• Absolute and relative EEG power are widely used but inconsistently applied.
• We conceptually and empirically evaluate assumptions underlying relative power.
• Absolute and relative EEG power capture overlapping but distinct information.
• Relative power does not fully correct for non-neural variability.
• Reporting both power types will enhance reproducibility in developmental EEG.

## 1 Introduction

Resting electroencephalography (EEG) is a widely used, non-invasive method for studying brain function during infancy and early childhood. Collected while a child sits quietly or watches a neutral stimulus, resting EEG provides temporally precise, cost-effective insights into brain activity in the absence of a task. It has been used to chart trajectories of brain function (Anderson and Perone, 2018) and to elucidate neural processes underlying cognitive, language, and socioemotional development (Benasich et al., 2008, Cuevas and Bell, 2022, Saby and Marshall, 2012). Beyond mapping typical development, resting EEG is sensitive to environmental influences (Bernier et al., 2016, Elansary et al., 2022, Gilbreath et al., 2023, Kling et al., 2023, Sandre et al., 2024, Troller-Renfree et al., 2020) and may help identify early neural markers of risk for adverse developmental and mental health outcomes (Brito et al., 2016, Brito et al., 2019, Gou et al., 2011, McLaughlin et al., 2010, Scher et al., 1996, Tan et al., 2023, Williams et al., 2012). Emerging evidence also indicates that resting EEG can be modified through early intervention (Chan and Ouyang, 2024, Debnath et al., 2020, Rockers et al., 2023, Troller-Renfree et al., 2022, Welch et al., 2022), making it a promising target for promoting positive developmental outcomes. With growing translational relevance, resting EEG is now incorporated into large-scale studies of child development (e.g., ECHO, HBCD, HBN; Alexander et al., 2017; Blaisdell et al., 2022; Fox et al., 2024) and increasingly adapted for mobile (Bhavnani et al., 2022, Troller-Renfree et al., 2021) and low-resource settings (Chin et al., 2025, Jensen et al., 2021, Neto et al., 2021). Resting EEG thus represents a powerful tool for expanding developmental research and improving the inclusion of historically underrepresented populations (Adams et al., 2024, Margolis et al., 2025). As the field increasingly relies on EEG to inform science and practice, methodological clarity and consistency are essential for producing interpretable, generalizable, and reproducible findings.

In developmental EEG research, a fundamental methodological decision involves how to quantify power, which is either in absolute or relative units. Absolute power refers to the raw magnitude of neural oscillatory activity within a given frequency band, typically expressed in microvolts squared per hertz (µV²). In contrast, relative power expresses each band’s contribution as a proportion of the total EEG power across all frequencies, yielding values between 0 and 1. Typically, when relative power is summed across bands, it equals 1. Based on their definition alone, absolute and relative power likely reflect different neurological processes, but both are currently widely used with little theoretical distinction.

Historically, relative power was developed to mitigate the influence of non-neural factors—such as skull thickness, hair texture, or electrode impedance—that can affect absolute power (Adams et al., 2024, Budzynski et al., 2009, Marshall et al., 2002, Nishiyori et al., 2021, Troller-Renfree et al., 2023). By normalizing across total power, relative power is thought to place individuals of different ages, head sizes, and recording systems on a comparable scale. It has also been argued to be more sensitive to developmental shifts in rhythm dominance, revealing proportional transitions from slower (e.g., theta) to faster frequencies as the brain matures (higher alpha/beta/gamma percentage; e.g., Barriga-Paulino et al., 2011; Cuevas and Bell, 2022; Marshall et al., 2002). However, these assumptions have rarely been tested directly. Despite the long-standing view that relative power “corrects” for non-neural variability, surprisingly few studies have examined whether it is, in fact, less sensitive than absolute power to these influences—or whether the two indices simply capture different aspects of the same underlying signal. Rather than treating relative power as a superior metric, we critically examine these assumptions and evaluate what relative power may contribute beyond absolute measures.

These considerations are especially important in developmental EEG research. While absolute and relative power are also used in adult EEG studies, debates about their relative merits are less central, in part because adult protocols are more standardized (e.g., gel electrodes, controlled impedance, reduced movement) and adult participants generally provide higher-quality recordings. Studies in adult samples may report and compare both metrics, but methodological discussions about when or why to use one over the other are not as prominent as in pediatric research. In contrast, developmental EEG is shaped by rapid behavioral and neurophysiological change and faces practical recording challenges, including frequent movement, variable compliance, fluctuating states (e.g., sleepiness, affect), and anatomical differences such as skull thickness, all of which introduce additional non-neural sources of variability (Georgieva et al., 2020, Noreika et al., 2020, Van der Velde and Junge, 2020). These factors motivated our focus on pediatric samples to critically evaluate what relative versus absolute power measures capture during early brain development.

## 2 Revisiting the role of relative power

Given the widespread use of both measures and the lack of consensus about their interpretation, the key question is not *which metric is better* but rather, *what does relative power actually buy us?* Despite its strengths, such as normalization and comparability, relative power has limitations. First, all frequency bands become inextricably linked once they enter relative space because each band’s value is a fraction of a shared total. In other words, if one band’s relative power rises, at least one other must fall, even if that other band’s absolute power did not change. This mathematical coupling can distort physiological interpretation. For example, a decrease in relative alpha power might reflect increased slower oscillations rather than reduced alpha activity. Second, total-power normalization combines band-limited oscillatory activity with the aperiodic 1/f background. Developmental changes in this broadband activity (e.g., flattening of the 1/f slope as the cortex matures) can spuriously inflate or reduce relative band measures, confounding true neural changes (Donoghue, Dominguez, et al., 2020). Together, these issues highlight that relative power is not a simple correction for amplitude-related variability, but a distinct representation of EEG spectral composition with its own interpretive challenges.

## 3 Current practices and field-level inconsistencies

Recent work underscores how inconsistently absolute and relative power are applied across developmental EEG research. A survey asked developmental EEG researchers to preregister their reporting of EEG power on the same hypothetical dataset. In the preregistration survey of 66 experts, just over half reported they would analyze both absolute and relative power in the same dataset, but there was little agreement on which to prioritize in main analyses versus supplemental materials (Troller-Renfree et al., 2025). About 41% said they would favor relative power, while 31% preferred absolute power for their primary findings. However, a systematic review of 56 developmental EEG studies in the top six journals publishing developmental EEG research found that published reports slightly favor absolute (70%) over relative (45%) power (percentages sum to over 100% as some manuscripts report both), although relative power is becoming increasingly common in recent years (Gray et al., 2026). Even within individual studies, the two measures are often used interchangeably for the same theoretical questions (Brown et al., unpublished results), despite their distinct interpretive basis. Such heterogeneity complicates cross-study comparison and the identification of reproducible neural biomarkers—especially in infancy and early childhood, when EEG properties change rapidly and differ substantially from adult patterns (Rico-Picó et al., 2023, Sandre et al., 2025). Clarifying what information each metric provides, and what it may obscure, is therefore essential for advancing developmental EEG research.

## 4 Goal of the present commentary

The goal of this commentary is to reexamine the assumptions underlying the use of relative power in developmental EEG and to empirically evaluate what, if anything, it “buys us” beyond absolute power. We compare absolute and relative power across canonical frequency bands and across multiple potential sources of variance in pediatric samples to identify where the two measures converge and where they diverge. We begin by examining *cross-power relations* between absolute and relative metrics, establishing the degree to which they capture shared versus distinct variance. We then evaluate whether relative power offers advantages in handling non-neural and state-related influences, including hair texture and thickness, affect, fatigue, and testing conditions (i.e., time of day). Together, these analyses test whether relative power provides unique developmental insight beyond absolute power or simply reframes the same underlying signal. By combining empirical examples with conceptual discussion, we aim to clarify the interpretive value—and limitations—of relative power for understanding early brain development.

## 5 Challenges in EEG measurement: empirical examples

### 5.1 Cross-power relations

Although absolute and relative power are often treated as interchangeable indices of EEG activity, the cross-power relations across multiple studies demonstrate that they capture sometimes overlapping, but not equivalent, information (See Table 1, data are from the following samples/studies: Brown et al., unpublished results; Sandre et al., 2025; Troller-Renfree et al., 2020; Troller-Renfree et al., 2026). For instance, absolute power reflects global power strength, with individuals high in one band tending to be high across all others. Relative power, by contrast, is inherently competitive: because it normalizes each band to the whole spectrum, increases in one rhythm necessarily reduce the apparent contribution of others, even when their absolute amplitude is unchanged. This means that relative metrics tend to show a negative correlation between lower frequency and higher frequency bands. Interestingly, higher frequency bands like gamma share high mutual information with an average correlation of greater than .8 across absolute and relative measures, while absolute and relative theta activity have a correlation of near zero (and, indeed, are negatively associated in some studies; for further discussion see supplement to Troller-Renfree et al., 2025).

**Table 1:** Mean correlation between absolute and relative power measures controlling for age across six independent samples and ten ages (all between 0 and 4 years; N > 3000).

*Note.* Blue shading with italics represents within-power associations (e.g., absolute power with absolute power), while orange shading with underlines shows associations between the same frequency band across power types (e.g., absolute theta with relative theta). Age was corrected by saving residuals from a linear regression model with age regressed on correlation values.

For developmental research, these associations are not trivial. While both absolute and relative power detail maturational changes in brain rhythms, they do so from different vantage points: absolute power is more likely to track the overall strengthening or attenuation of oscillatory generators with age, whereas relative power emphasizes the shifting dominance from slow to fast rhythms. The cross-power associations may highlight the need for differing hypotheses—particularly in the theta band where it may be prudent to have different or even opposing hypotheses across power types.

### 5.2 Hair texture and thickness

A growing body of work suggests that differences in hair texture and thickness may impact power, but few studies have examined relations between hair and both absolute and relative power (Adams et al., 2024, Etienne et al., 2020, Lees et al., 2024). One may imagine, for instance, that relative power may handle this confound if hair-related power associations are present in both band and total power. To test this, we examined associations between absolute and relative power from 52 sociodemographically diverse 3.5-year-old children (PI: Troller-Renfree with data collection ongoing. Sample drawn from Troller-Renfree et al. (2025) [N = 24] and additional families from the community [N = 28] during the lights-off condition of a resting EEG paradigm and mothers’ ratings of their child’s hair texture (straight, wavy, curly, kinky/coily) and hair thickness (thin, medium, thick). Analyses showed that hair texture, but not thickness, was associated with EEG power in the gamma band, and this was true for both absolute (*r* = -.287, *p* = .039) and relative power (*r* = -.246, *p* = .078), although associations with relative power fell in the statistical margin. No significant associations were observed in other bands. Together, these data suggest that hair texture is associated with EEG power in higher frequency bands and that this signal is detectable in both absolute and relative power.

### 5.3 State-related and contextual influences

**Fig. 1:**
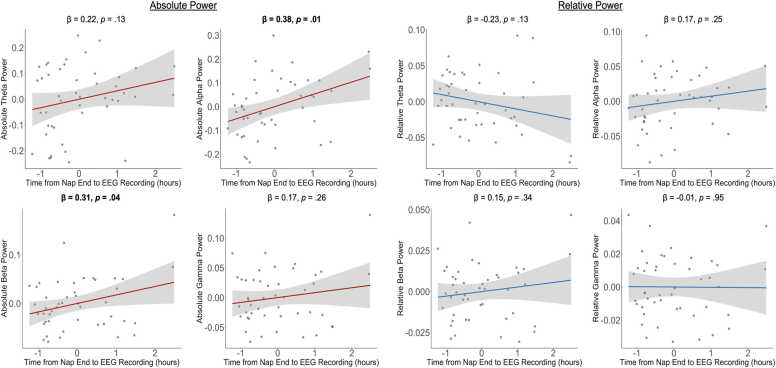
*Partial regression plots depicting associations between time from nap end to the start of EEG recording (an index of fatigue) and absolute and relative EEG power in infants. Note.* Data are from 46 12-month-old infants who completed a 3-minute resting-state EEG recording while watching a silent video of moving lights and objects, including infants who napped. Analyses controlled for infant age (in weeks). Bolded values indicate significant associations. Time from nap end to EEG recording was calculated as the difference (in hours) between when mothers reported their infant’s most recent nap ended and when the EEG recording began. Absolute and relative EEG power were computed from log10-transformed values and averaged across electrodes (whole-brain, excluding the outermost ring). See Sandre et al. (2025) for full preprocessing details.

**Fig. 2:**
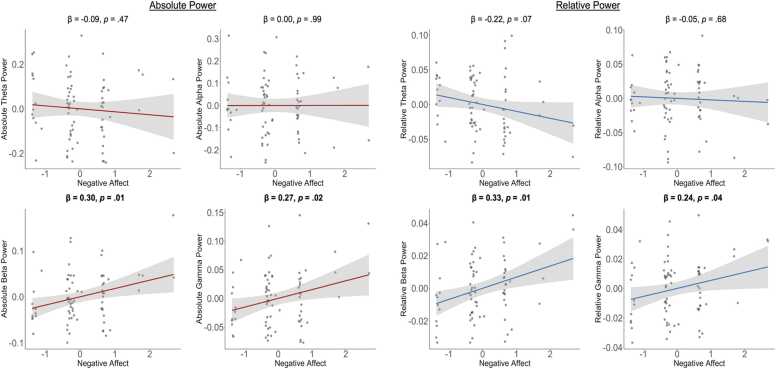
*Partial regression plots depicting associations between negative affect and absolute and relative EEG power in infants. Note.* Data are from 74 12-month-old infants who completed a 3-minute resting-state EEG recording while watching a silent video of moving lights and objects. Analyses controlled for infant age (in weeks). Bolded values indicate significant associations. Negative affect was assessed immediately before EEG recording, when mothers were asked: “How often has your infant shown signs of being in a negative mood today? This includes crying, whimpering, fussing, frowning, screaming, or showing sadness, fear, or anger.” Responses were provided on a 5-point scale (1 = Not at all, 2 = Rarely, 3 = Sometimes, 4 = Often, 5 = Very often). Absolute and relative EEG power were computed from log10-transformed values and averaged across electrodes (whole-brain, excluding the outermost ring). See Sandre et al. (2025) for full preprocessing details.

**Fig. 3:**
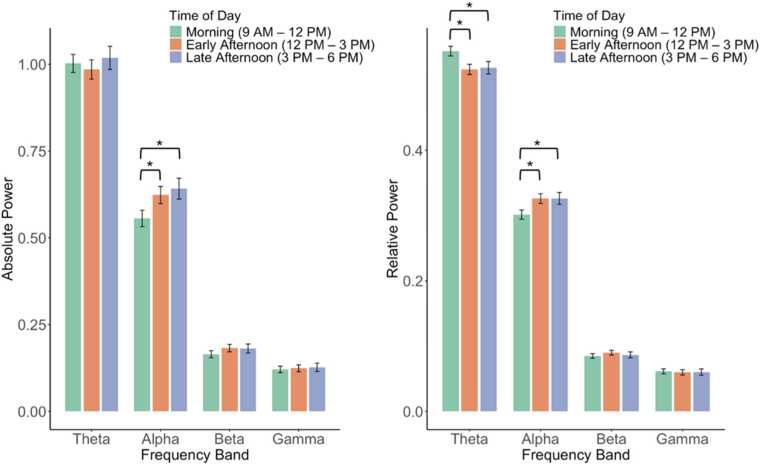
*Associations between time of day of EEG recording and absolute and relative EEG power in infants. Note.* Data are from 74 12-month-old infants who completed a 3-minute resting-state EEG recording while watching a silent video of moving lights and objects. Bars represent estimated marginal means of EEG power, adjusted for infant age (in weeks). Error bars indicate ±1 standard error of the estimated marginal mean. *p < .05. Time-of-day groups included Morning (n = 30), Early Afternoon (n = 26), and Late Afternoon (n = 18). Absolute and relative EEG power were computed from log10-transformed values and averaged across electrodes (whole-brain, excluding the outermost ring). See Sandre et al. (2025) for full preprocessing details.

EEG power is sensitive to a variety of state-related factors, such as arousal, affect, and movement (Georgieva et al., 2020, Van der Velde and Junge, 2020). Contextual factors such as time of day may also be an especially relevant source of variability in EEG power during infancy and childhood, when sleep–wake cycles and circadian rhythms are still developing (Thomas et al., 2014). Relative power is often presumed to be less sensitive to such influences because it normalizes across total activity, but few studies have directly tested this assumption. To examine this, we compared associations of fatigue, affect, and time of day with both absolute and relative power in 12-month-old infants drawn from Sandre et al. (2025). Analyses showed that fatigue, indexed by time since the last nap, was associated with greater absolute alpha and beta power but not relative power (see Fig. 1), suggesting that relative power is less affected by band-specific increases in signal amplitude. In contrast, higher negative affect was linked to increases in both absolute and relative beta and gamma power (see Fig. 2), indicating that both metrics capture affective arousal. Time of day also mattered (see Fig. 3): infants tested in the early and late afternoon showed higher absolute alpha power than those tested in the morning, whereas relative power showed higher theta and lower alpha in the morning. Together, these findings indicate that relative power is somewhat—but not entirely—insensitive to transient state or contextual factors, underscoring that it does not universally correct for these sources of variability.

## 6 Implications for the field

Absolute and relative power provide complementary, rather than interchangeable, perspectives on developmental EEG. Cross-power correlations indicate that higher-frequency bands, like gamma, tend to align across measures, whereas lower-frequency bands, like theta, may show weak or even negative associations, pointing to potentially distinct functional or developmental interpretations. Relative power is not a universal safeguard against variability: while it may dampen some global amplitude effects (e.g., fatigue), it remains sensitive to affect, time of day, and anatomical factors such as hair texture. Nonetheless, it captures proportional shifts across frequency bands, revealing maturational or functional patterns that may be less apparent when relying solely on absolute power.

## 7 Recommendations for the field

To advance developmental EEG research and ensure findings are interpretable and comparable, we offer several field-level practices informed by these insights. First, we suggest researchers preregister and justify their use of either absolute or relative power based on existing literature and/or meta-analyses. Alternatively, researchers should report both absolute and relative power whenever possible (even if one is supplemental), as the two metrics capture overlapping but sometimes distinct information. To mitigate the impact of additional comparisons when reporting both metrics, researchers can apply multiple-comparison corrections (e.g., false discovery rate) or use multivariate approaches (e.g., MANOVA, mixed-effects models). Data-reduction methods, such as latent profile analysis, may also be useful to reduce the number of comparisons (e.g., Pierce et al., 2019).

Second, researchers should transparently document and justify methodological decisions, including frequency band definitions and preprocessing steps, to support reproducibility and clarify how analytic choices may influence results. For example, when computing relative power with a log transformation, it is important to report whether absolute power was log-transformed prior to relative power computation. Examining additional data from a recent systematic review (Gray et al., 2026) and other sources, it appears that more studies log-transform before relative power calculation (e.g., Brito et al., 2022; Debnath et al., 2020; Jensen et al., 2021; Vanderwert et al., 2016), while other studies log-transform after calculating relative power (e.g., Bick et al., 2022; Brito et al., 2020; Cragg et al., 2011), which results in a negative relative power value as a log of a decimal is negative. Log-transforming before computing relative power changes the scale non-linearly, so the resulting proportions reflect fractions of log-transformed rather than raw power, which can change the relative rank of power values in extreme cases. In contrast, log transforming after relative power results in values that are negative and no longer sum to 1, reducing the interpretability of these values and the relations to each other. In our experience, both methods produce highly similar relative values within each frequency band (*r*s > .9), suggesting that choosing one over the other is unlikely to meaningfully impact investigations of developmental changes or individual differences. This choice does, however, affect the scale of values and reproducibility, so explicitly reporting the method enhances transparency, interpretability, and comparability across studies. Similarly, there are many other processing decisions that may have more significant impacts on the alignment of absolute and relative power values, which merit additional investigation (see Troller-Renfree et al. 2025 for further discussion).

Third, assess factors such as hair texture, fatigue, affective state, and contextual variables like time of day to evaluate their influence on absolute and relative EEG power. While researchers cannot—or need not—control for every influence, identifying those with the strongest impact allows for practical adjustments, such as testing infants after a nap when feasible, and/or including these factors as covariates in analyses. To support this effort, we provide in the Supplementary Materials a brief questionnaire (available in English and Spanish) that collects parent-report information on child state-related factors (e.g., sleepiness, hunger, hours slept the night before, time since last meal or nap, positive and negative affect) and experimenter-report information on contextual variables (e.g., time of day of recording).

## 8 Future directions

Future research should continue to delineate the independent and complementary aspects of absolute and relative power. In particular, it will be important to examine how biological characteristics (e.g., hair texture, skull thickness), state-related factors (e.g., fatigue, affect, time of day), and experimental or methodological factors (e.g., task context, reference scheme, preprocessing choices) may differentially influence absolute and relative power. Identifying the conditions under which each measure provides distinct or convergent information will guide more consistent metric selection, reporting, and interpretation across developmental EEG studies. For instance, relative power has been proposed to correct for differences in skull thickness, but this has not been systematically examined across development, particularly at times when the skull is changing rapidly, such as when the fontanelle is closing. Thus, it remains unclear whether total-power normalization meaningfully controls for such anatomical differences. Similarly, a systematic evaluation of whether relative power may better handle differences due to electrode impedance and signal quality has not been conducted during development.

Our analyses did not separate periodic and aperiodic components of the EEG signal, meaning that the relations we observed between absolute and relative power may differ if oscillatory activity were examined in isolation from the aperiodic background (Ostlund et al., 2022, Rico-Picó et al., 2023). Future work using parameterization methods could clarify these associations and determine whether patterns of correspondence between absolute and relative metrics remain similar when aperiodic influences are removed (Donoghue, Haller, et al., 2020).

In addition, future studies may consider leveraging simulated EEG data with known signal amplitude and noise parameters to clarify the mathematical behavior of absolute and relative power under various conditions, isolating when relative power may or may not be more reflective of underlying neural activity.

Establishing normative reference datasets that encompass both absolute and relative power across diverse populations will also be crucial for harmonization efforts and for situating individual differences within the context of typical, generalizable neurodevelopmental trajectories. For example, similar efforts in structural MRI (Alldritt et al., 2024, Bethlehem et al., 2022) have shown that harmonizing data across different scanners can produce meaningful centile scores applicable across datasets and samples. With rigorous harmonization and data integration, such approaches may also prove fruitful for resting EEG.

Finally, although the current manuscript focuses on pediatric resting EEG, evaluating whether similar patterns hold in task- or stimulus-based EEG paradigms represents an important avenue for future research.

## 9 Conclusion

Accurate and consistent measurement of EEG power is essential for advancing developmental neuroscience. This commentary reexamines the assumption that relative power simply corrects for non-neural variability, showing instead that it provides a distinct and complementary perspective on the EEG signal. Recognizing the unique strengths and limitations of both absolute and relative measures allows researchers to select and interpret these metrics more deliberately, grounded in their theoretical and methodological implications. As resting EEG becomes increasingly common in large-scale developmental studies, standardizing analytic and reporting practices will be critical for ensuring findings are valid, reproducible, and comparable. Such efforts will strengthen EEG’s interpretive value for understanding early brain development and its utility in identifying mechanisms of risk and resilience across diverse populations.

## CRediT authorship contribution statement

**Aislinn Sandre:** Writing – review & editing, Writing – original draft, Investigation, Formal analysis, Conceptualization. **Sonya V. Troller-Renfree:** Writing – review & editing, Writing – original draft, Investigation, Formal analysis, Conceptualization.

## Declaration of Competing Interest

The authors declare that they have no known competing financial interests or personal relationships that could have appeared to influence the work reported in this paper

## Data Availability

Data will be made available on request.

## Footnotes

Appendix A Supplementary data associated with this article can be found in the online version at doi:10.1016/j.dcn.2026.101698.
