# Supplementary material for: Power struggles: Absolute vs. relative EEG power in developmental neuroscience
Source: Dev Cogn Neurosci. 2026 Feb 26;79:101698. doi: 10.1016/j.dcn.2026.101698 (PMC12993156; doi:10.1016/j.dcn.2026.101698)
Supplement: Supplementary file 1 — Supplementary material [file mmc1.docx]

**Power Struggles: Absolute vs. Relative EEG Power in Developmental Neuroscience**

***Supplementary Materials***

**Child State and Context Questionnaire**

Participant ID / ID del participante: ___________

Date & Time of Administration / **Fecha y hora de administración:** (MM/DD/YY) (HH:MM)

**Instructions / Instrucciones:**
The following questions should be asked **immediately before capping the participant** (i.e., on the day of their lab visit). / Las siguientes preguntas deben hacerse **inmediatamente antes de colocar el gorro de EEG** (es decir, el día de la visita al laboratorio).

**Introduction / Introducción**

I have a few questions about [child_name]'s sleep and eating patterns, as well as their mood today. / Tengo algunas preguntas sobre los patrones de sueño y alimentación de [Nombre del niño/a], y su estado de ánimo hoy.

**Sleep/ Sueño**

1. In a typical week, how many hours does [child_name] sleep at night? (HH:MM) / En una semana típica, ¿cuántas horas duerme [Nombre del niño/a] en la noche? (HH:MM)
2. How many hours did [child_name] sleep last night? (HH:MM) / ¿Cuántas horas durmió [Nombre del niño/a] anoche? (HH:MM)
3. What time did [child_name] wake up today? (HH:MM) / ¿A qué hora se despertó hoy [Nombre del niño/a]? (HH:MM)
4. What time did [child_name] last take a nap today? (HH:MM) If no nap has been taken, enter 00:00. / ¿A qué hora [Nombre del niño/a] tomó una siesta hoy? (HH:MM) Si no se ha tomado ninguna siesta, ingrese 00:00]
5. How long did [child_name] nap for? (HH:MM) / ¿Cuánto tiempo durmió la siesta [Nombre del niño/a]? (HH:MM)

1. On a scale from 1 (not tired at all) to 5 (very tired), how tired is [child_name] currently? / En una escala del 1 (nada cansado) al 5 (muy cansado), ¿qué tan cansado está [Nombre del niño/a] actualmente?

**Hunger/ Hambre**

1. Has [child_name] eaten today? / ¿Ha comido [Nombre del niño/a] hoy?

☐ Yes / Sí

☐ No / No

1. What time did [child_name] last eat? (HH:MM) If child hasn't eaten, please enter 00:00. / ¿A qué hora comió por última vez [Nombre del niño/a]? (HH:MM) Si el niño o la niña no ha comido, por favor ingrese 00:00
2. On a scale from 1 (not hungry at all) to 5 (very hungry), how hungry is [child_name] currently? / En una escala del 1 (no tiene hambre) al 5 (tiene mucha hambre), ¿cuánta hambre tiene [Nombre del niño/a] actualmente?

**Affect / Afecto**

1. How often has [child_name] showed signs of being in a positive mood today? This includes smiling, laughing, cooing, squealing, or showing happiness, joy, or enthusiasm. Would you say: / ¿Con qué frecuencia [Nombre del niño/a] mostró signos de estar de buen humor hoy? Esto incluye sonreír, reír, arrullar, chillar o mostrar felicidad, alegría o entusiasmo. Dirías que:

Answer / Respuesta (check one / marque uno):

☐ Not at all / Para nada

☐ Rarely / Raramente

☐ Sometimes / A veces

☐ Often / Con frecuencia

☐ Very often / Con mucha frecuencia

1. How often has [enrollment_arm_1][child_name] showed signs of being in a negative mood today? This includes crying, whimpering, fussing, frowning, screaming, or showing sadness, fear, or anger. Would you say: / ¿Con qué frecuencia [Nombre del niño/a] ha mostrado signos de estar de mal humor hoy? Esto incluye llorar, gemir, quejarse, fruncir el ceño, gritar o mostrar tristeza, miedo o enojo. Dirías que:

Answer / Respuesta (check one / marque uno):

☐ Not at all / Para nada

☐ Rarely / Raramente

☐ Sometimes / A veces

☐ Often / Con frecuencia

☐ Very often / Con mucha frecuencia
